# The Templar’s Magazine

**Published:** 1862-07

**Authors:** 


The Templar's Magazine.—This journal, devoted to the cause of temperance, and edited by J. Wadsworth and M. P. Gaddis, is before us. It is doing a noble work in a noble cause, a great humanitarian work. This matter of intemperance has, probably, produced more misery, poverty and wretchedness than any other one thing that has ever existed in our country, and yet it is regarded by almost every body as a small matter, at least practically, and by the great mass of the people is overlooked entirely, and by a great many sustained.

Any one who espouses the cause of temperance has an almost overwhelming, surging tide of opposition to contend with. This journal takes a strong stand upon this subject, and right manfully does it battle, too.

This subject is one fraught with fearful interest at this time; the minds of the people are so much absorbed with other things, that the subject of temperance is wholly passed by, and intemperance is running riot, with a higher hand than ever before. It is under such circumstances that we find the Templar's Magazine doing its work. We hope it will go on, and not be discouraged though it works in darkness, and that it shall soon see the fruits of its labors in the moral renovation of the world.

It should be in every family in the land. It is published by J. Wadsworth, at $1.00 per annum, in advance. May its circulation be largely increased. T.

